# Identification of microRNAs That Regulate TLR2-Mediated Trophoblast Apoptosis and Inhibition of IL-6 mRNA 

**DOI:** 10.1371/journal.pone.0077249

**Published:** 2013-10-15

**Authors:** Manish Garg, Julie A. Potter, Vikki M. Abrahams

**Affiliations:** Department of Obstetrics, Gynecology & Reproductive Sciences, Divisions of Reproductive Sciences and Maternal-Fetal Medicine, Yale School of Medicine, New Haven, Connecticut, United States of America; Otto-von-Guericke University Magdeburg, Germany

## Abstract

While infection-induced placental inflammation is a common mechanism of adverse pregnancy outcome, some pathogens can also trigger placental apoptosis, and Toll-like receptors (TLRs) mediate this response. Treatment of human first trimester trophoblast cells with bacterial peptidoglycan (PDG) reduces their constitutive secretion of IL-6 protein and induces apoptosis. This apoptotic response is dependent upon the cell’s expression of TLR1, TLR2 and TLR10, and their lack of TLR6, such that ectopic expression of TLR6 prevents PDG-induced apoptosis and restores IL-6 production. In this current study we have identified three microRNAs (miRs) that regulate TLR2-mediated responses in the human trophoblast. Herein we report that miR-329 plays a pivotal role in mediating PDG-induced trophoblast apoptosis and inhibition of IL-6 mRNA expression by targeting the NF-κB subunit, p65. TLR2 activation by PDG upregulates miR-329 expression and inhibits NF-κB p65 and IL-6 mRNA, and this is reversed by the presence of TLR6. Moreover, inhibition of miR-329 prevents PDG-induced inhibition of NF-κB p65 and IL-6 mRNA expression, and restores cell survival. In addition, we have found miR-23a and let-7c to directly regulate PDG-mediated inhibition of IL-6 mRNA. TLR2 activation by PDG upregulates miR23a and let-7c expression and this is reversed by the presence of TLR6. Furthermore, inhibition of both miR23a and let-7c prevents PDG-inhibition of trophoblast IL-6 mRNA expression. Together, our findings suggest that multiple miRs are involved in the molecular regulation of TLR2-mediated responses in the trophoblast towards gram-positive bacterial components.

## Introduction

An intrauterine infection can threaten fetal well-being and pregnancy outcome by gaining access to gestational tissues, such as the placenta, and by triggering an immune response [1]. There is a strong clinical correlation between bacterial infections and preterm birth [2]; and other complications of pregnancy, like preeclampsia, may also have an underlying infectious element [3]. The mechanism by which an infection can lead to adverse pregnancy outcome is thought to involve innate immune responses towards the pathogen, leading to excessive inflammation at the maternal-fetal interface [1], and studies focusing on the pathways involved have implicated pattern recognition receptors (PRR), such as the Toll-like receptors (TLRs), as playing an important role [1,4]. Through TLRs, the placental trophoblast cells sense and respond to a variety of infectious stimuli [4]. Moreover, using specific TLR agonists and TLR-deficient mice, these PRRs are now known to be involved in the pathogenesis of infection-associated prematurity and pregnancy complications [4-7]. While inflammation is a frequent and common mechanism of TLR function in the trophoblast and adverse pregnancy outcome [1], excessive placental apoptosis has also been associated with abnormal pregnancies [8,9]. Indeed, administration of gram-positive bacterial peptidoglycan (PDG) to pregnant mice, rather than inducing inflammation, triggers placental apoptosis [4] and preterm labor [6], as does the gram-positive bacterium, Group B Streptococcus [10]. Moreover, TLR2 has been shown to mediate this PDG-induced response in the trophoblast [4,11]. 

Upon ligand sensing, TLR2 functions by either homodimerizing, or heterodimerizing with its co-receptors, TLR1, TLR6 or TLR10 [12-14]. Human first trimester trophoblast cells express TLR1, TLR2 and TLR10, but lack TLR6 [4,11,15]. Following exposure to the TLR2 agonist, PDG, these trophoblast cells undergo apoptosis, and in parallel, their constitutive NF-κB activity and basal secretion of IL-6 protein is reduced [4,11]. This apoptotic response is mediated by TLR1, TLR2 and TLR10 [4,15]. Moreover, it is the absence of TLR6 that confers the cell's sensitivity to PDG, such that overexpression of this co-receptor prevents PDG-induced apoptosis and restores constitutive chemokine production [4]. While these findings demonstrate a role for TLR6 as a regulatory switch than can control TLR2-mediated trophoblast apoptosis and cell survival, questions regarding the molecular mechanisms involved still remain.

One way in which TLR signaling can be regulated is through microRNAs (miRNA); non-coding small RNAs that regulate gene expression by either destabilizing or inhibiting the translation of target mRNAs at the post-transcriptional level [16,17]. Activation of TLRs can induce miRNAs, which in turn regulate TLR-mediated responses [17]. Recently miRNAs have been shown to play a role in the regulation of normal labor [18,19], the regulation of normal trophoblast function [20-24]; and altered miRNA expression patterns at the maternal-fetal interface have been associated with prematurity [18,25,26]. Therefore, changes in the expression and function of these intracellular regulators of inflammatory responses and processes may play a critical role in the pathogenesis of infection-associated adverse pregnancy outcome. However, while recent studies have demonstrated altered miRNA expression in hypoxic term trophoblast, trophoblast cells lines exposed to environmental toxins or LPS, or in placenta from pathological pregnancies [18,25-32], nothing is known about their role in the regulation of TLR2-mediated responses to bacterial PDG in the trophoblast. In this current study we have identified miR-329 as playing a pivotal role in mediating PDG-induced trophoblast apoptosis and inhibition of IL-6 expression by targeting the NF-κB subunit, p65. In addition, we have identified, miR-23a and let7c, as playing a role in regulating PDG-mediated inhibition of trophoblast IL-6 mRNA expression.

## Materials and Methods

### Cell culture and transfection

For this study, the SV40-transformed human first trimester trophoblast cell line, 3A, which lacks TLR6 (TLR6^-^), and the 3A cells stably transfected to express human TLR6 (TLR6^+^) were used [4]. Both cell lines were cultured at 37**°**C/5% CO_2_ in RPMI 1640 (Invitrogen; Grand Island, NY), supplemented with 10% FBS (HyClone; Rockford, IL), 10mM HEPES, 0.1mM MEM nonessential amino acids, 1mM sodium pyruvate, and 100nM penicillin/streptomycin (Invitrogen; San Diego, CA). The media used for the TLR6^+^ cells was also supplemented with 200μg/ml blasticidin (Invivogen). For all experiments, the TLR6^-^ and TLR6^+^ cells were treated with or without bacterial peptidoglycan (PDG) isolated from *Staplylococcus aureus* (Invivogen) in serum-free OptiMEM (Invitrogen). For some experiments the NF-κB activation inhibitors, 481406 or BAY 11-7082 (Calbiochem; Billerica, MA) were used. For transfection studies, TLR6^-^ cells were transfected with 100nM of either an anti-miR scramble sequence control or specific inhibitors of miR-329, miR-23a or Let-7c (Mirvana, Applied Biosystems; Grand Island, NY) using siPORT^TM^ NeoFX^TM^ (Invitrogen). For monitoring transfection efficiency, cells were transfected with Cy-3 labeled anti-miR scramble sequence and uptake was visualized by fluorescence microscopy.

### Quantitative Real Time RT-PCR

Total RNA was extracted using TRIzol (Life Technologies; Grand Island, NY). Briefly, 1ml of Trizol was added to adherent cells for 5min at 4°C. The cells were scrapped, vortexed to homogenize and incubated for 10min at 4°C. To the homogenized samples, 0.2ml of chloroform was added, vortexed and incubated for 10min. For phase separation the suspension was centrifuged at 13000xg for 15min. The aqueous phase was collected, RNA was precipitated, washed and then resuspended in nuclease free water. The total RNA concentration was measured using a NanoDrop. To measure the expression of IL-6, NF-κB p65, NFKB1 mRNA, 1μg of total RNA was reverse transcribed using Superscript II RT kit (Invitrogen). The primer sequences used were as follows: IL-6: forward (TGCAGAAAAAGGCAAAGAAT) and reverse (CTGACCAGAAGAAGGAATGC); NFκB p65: forward (tctgcttccaggtgacagtg) and reverse (ATCTTGAGCTCGGCAGTGTT); NFKB1: forward (CCTGAGACAAATGGGCTACAC) and reverse (TTTAGGGCTTTGGTTTACACGG). GAPDH served as a housekeeping gene: forward (GGGGAAGGTGAAGGTCGGAGT) and reverse (GGAGGGATCTCGCTCCTGGAA). For IL-6, p65 and GAPDH, 45 cycles of PCR was performed at 95°C for 10s, 50°C for 30s, 72°C for 30min. For NFKB1 and GAPDH, thermal cycle conditions were the following: 95°C for 10min, 40 cycles of 95°C for 15s and 60°C for 60s. The expression of miR-23a; miR23b; miR-149; miR-155; miR-329 and let-7c was measured using the Taqman MicroRNA Assay and normalized to the endogenous housekeeping miRNA-374 control (Applied Biosystems). Briefly, 10ng of total RNA was reverse transcribed by Taqman reverse transcriptase kit (Applied Biosystems) in a reaction volume of 15μl containing 3μl of primers specific for miR-23a; miR23b; miR-149; miR-155; miR-329; let-7c or miR-374 and thermal cycling conditions were 16°C for 30min, 42°C for 30min, 85°C for 5min. The cDNA was amplified using Taqman Universal PCR Master Mix II, with UNG and specific primers for miR-23a; miR23b; miR-149; miR-155; miR-329; let-7c or miR-374 using the following conditions: 50°C for 2min, 95°C for 10min, 95°C for 15sec and 60°C for 60sec. The data was analyzed using the Δ-Δ CT method and was plotted as fold change (FC) in the expression of gene of interest normalized to the housekeeping gene.

### MicroRNA microarray assay

To identify microRNAs that could potentially target NF-κB p65 mRNA or IL-6 mRNA, and that were differentially expressed in PDG treated TLR6^-^ and TLR6^+^ cells, three independent experiments were pooled and 5μg of total RNA sample from each treatment group was sent to LC Sciences (Houston, TX) for miRNA microarray analysis. The miRNA expression profile was performed using µParaflo microfluidic chip (LC Sciences) and analysis was performed using the human miRNA array (LC Sciences), which identifies miRNA transcripts based on miRBase Release 18.0 (http://www.sanger.ac.uk/Software/Rfam/miRNA/). After receiving the miRNA microarray data, we identified miRs that putatively target NF-κB p65 mRNA and IL-6 mRNA using microrna.org.

### Caspase-3 activity assay

As a measure of apoptosis, the CaspaseGlo assay (Promega; Madison, WI) was used to measure the activity of caspase-3 as previously described [4]. Briefly, 10μg of total cellular protein was incubated with caspase-3 substrate for 1h at room temperature in the dark, after which luminescence was measured using a TD-20/20 luminometer (Turner Designs; Sunnyvale, CA).  The data was recorded as relative light unit (RLU) in triplicates.

### Western Blot analysis

Proteins were diluted with gel loading buffer and boiled for 5 min, after which they were resolved on a 12% polyacrylamide gel under reducing conditions and then transferred to a PVDF membrane (PerkinElmer; Waltham, MA). Membranes were probed with antibodies against total NF-κB p65 (SC-8008, Santa Cruz Biotechnology; Santa Cruz, CA), NFKB1 (p105/p50; Clone H-119, SC-7178, Santa Cruz Biotechnology), or β-actin (A2066, Sigma Aldrich, St Louis, MO) followed by either a horse anti-mouse IgG or a goat anti-rabbit IgG secondary antibody conjugated to peroxidase (Vector Labs; Burlingame, CA). The peroxidase-conjugated antibody was detected by enhanced chemiluminescence (PerkinElmer). Images were recorded and semi-quantitative densitometry performed using the Gel Logic 100 and Carestream MI software (Carestream Molecular Imaging; Rochester, NY). 

### Statistical Analysis

All data sets are expressed as mean ± standard deviation (SD). All experiments were performed independently at least three times, and the cumulative data from three or more experiments was plotted and used for statistical analysis. For statistical significance (*p*<0.05), the Wilcoxon signed rank test was performed using Prism software (GraphPad Software, Inc).

## Results

### TLR6 prevents TLR2-mediated inhibition of trophoblast IL-6 mRNA expression

**Figure 1:**
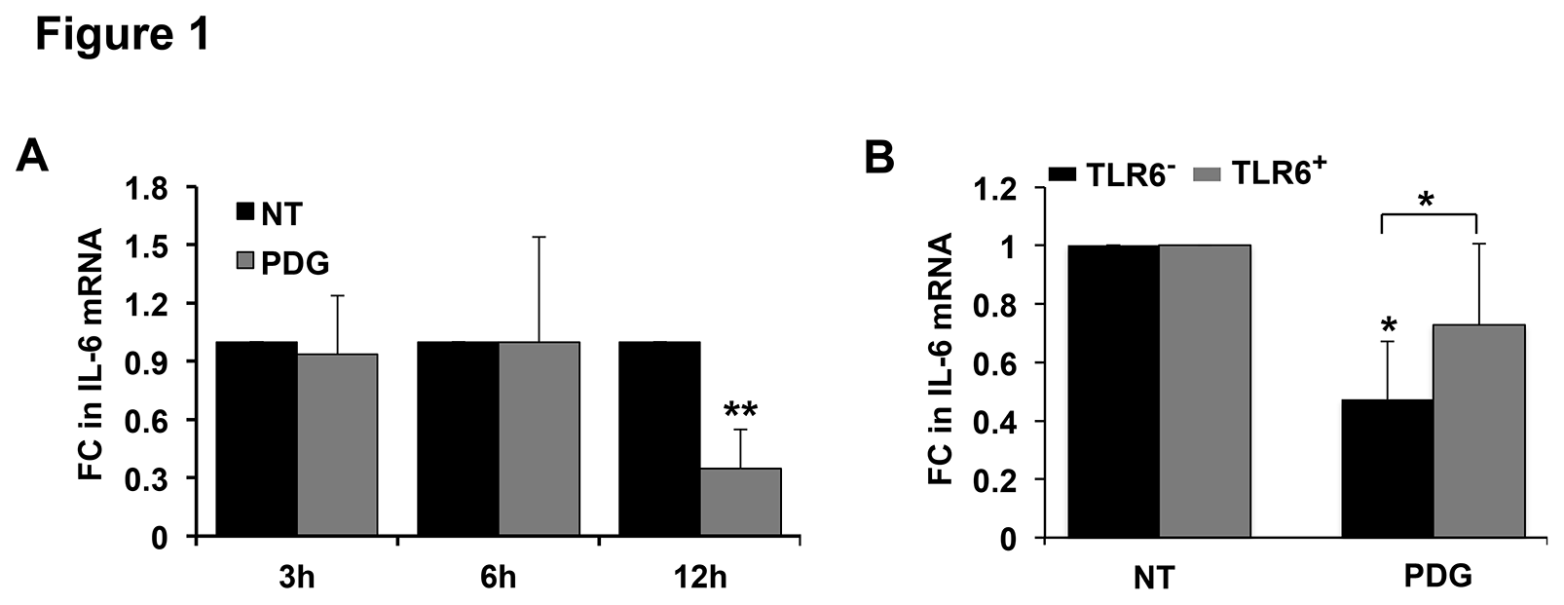
TLR6 restores PDG-mediated inhibition of trophoblast IL-6 expression. (A) The human first trimester trophoblast cell line, 3A, which lacks TLR6 (TLR6^-^), was treated with no treatment (NT) or peptidoglycan (PDG) at 80μg/ml for 3-24h, after which total RNA was isolated and IL-6 mRNA measured by qRT-PCR using GAPDH as an internal control (FC = fold change). After 12h, PDG treatment significantly reduced IL-6 mRNA levels (n=7). (B) Trophoblast cells either lacking TLR6 (TLR6^-^) or stably transfected to express TLR6 (TLR6^+^) were treated with NT or PDG (80μg/ml). After 12h, IL-6 mRNA was measured by qRT-PCR. The presence of TLR6 significantly reversed PDG-induced inhibition of IL-6 mRNA expression (n=5). *p<0.05, **p<0.001 relative to the NT control unless otherwise indicated.

We have previously demonstrated that human first trimester trophoblast cells expressing TLR1, TLR2 and TLR10, but lacking TLR6 (TLR6^-^) respond to bacterial PDG by undergoing apoptosis with concomitant reduced constitutive IL-6 protein secretion; and this response is reversed in the same cells if they are transfected to express functional TLR6 (TLR6^+^) [4,11,15]. To begin to understand the molecular mechanism by which TLR2 activation leads to this inhibition of IL-6 secretion, we first sought to determine whether this response was occurring at the transcriptional level. Treatment of TLR6^-^ trophoblast cells for 3h and 6h with PDG showed no significant changes in IL-6 mRNA expression levels when compared to the no treatment (NT) control.  However, at the later time point of 12h, PDG significantly inhibited IL-6 mRNA expression by 65.3 ± 20.4 % when compared to the NT control (Figure 1A). In contrast, treatment of TLR6^+^ trophoblast cells with PDG for 12h had no significant effect on basal IL-6 mRNA levels when compared to the NT control, and IL-6 mRNA levels in the PDG-treated TLR6^+^ cells were significantly higher than in the PDG-treated TLR6^-^ cells (Figure 1B). Thus, similarly to our observations at the protein level [4], the presence of TLR6 prevents the ability of PDG to reduce constitutive trophoblast IL-6 production at the transcriptional level. 

### Inhibition of NF-κB reduces IL-6 expression and triggers apoptosis in first trimester trophoblast

**Figure 2:**
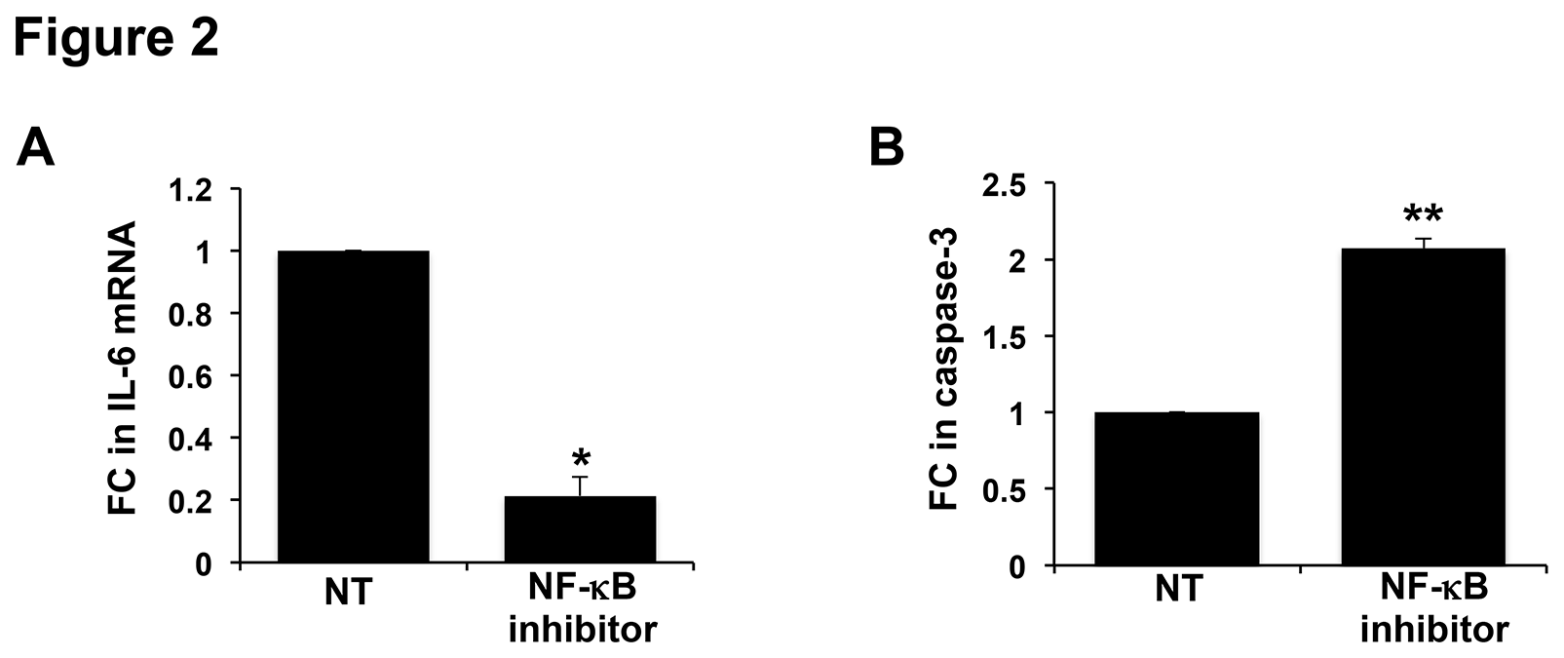
Inhibition of trophoblast NFκB activity reduces IL-6 mRNA expression and induces apoptosis. TLR6^-^ trophoblast cells were treated with no treatment (NT) or a NF-κB inhibitor. (A) After 12h treatment with the NF-κB inhibitor, IL-6 mRNA levels were significantly reduced (n=5). (B) After 24h, the NF-κB inhibitor significantly elevated caspase-3 activity (n=4). *p<0.05; **p<0.001 relative to the NT control.

We have previously reported that treatment of TLR6^-^ trophoblast cells with PDG reduces basal NF-κB activity levels using a luciferase reporter assay, and this was restored by the presence of TLR6 [4]. Therefore, we hypothesized that NF-κB regulates TLR2-mediated apoptosis and IL-6 production in the trophoblast. To test this, TLR6^-^ cells were treated with an NF-κB inhibitor at a concentration that blocks NFκB activity in different cell types [33,34], after which IL-6 mRNA and caspase-3 activity (a marker of apoptosis) were measured. As shown in Figure 2A, after 12h, the NF-κB inhibitor significantly reduced basal trophoblast IL-6 mRNA levels by 78.6 ± 5.5% compared to the NT control, and after 24h, the NF-κB inhibitor significantly elevated trophoblast caspase-3 activity by 2.1 ± 0.2 fold compared to the NT control cells (Figure 2B).

### PDG inhibits trophoblast NF-κB p65 expression and this is reversed by the presence of TLR6

**Figure 3:**
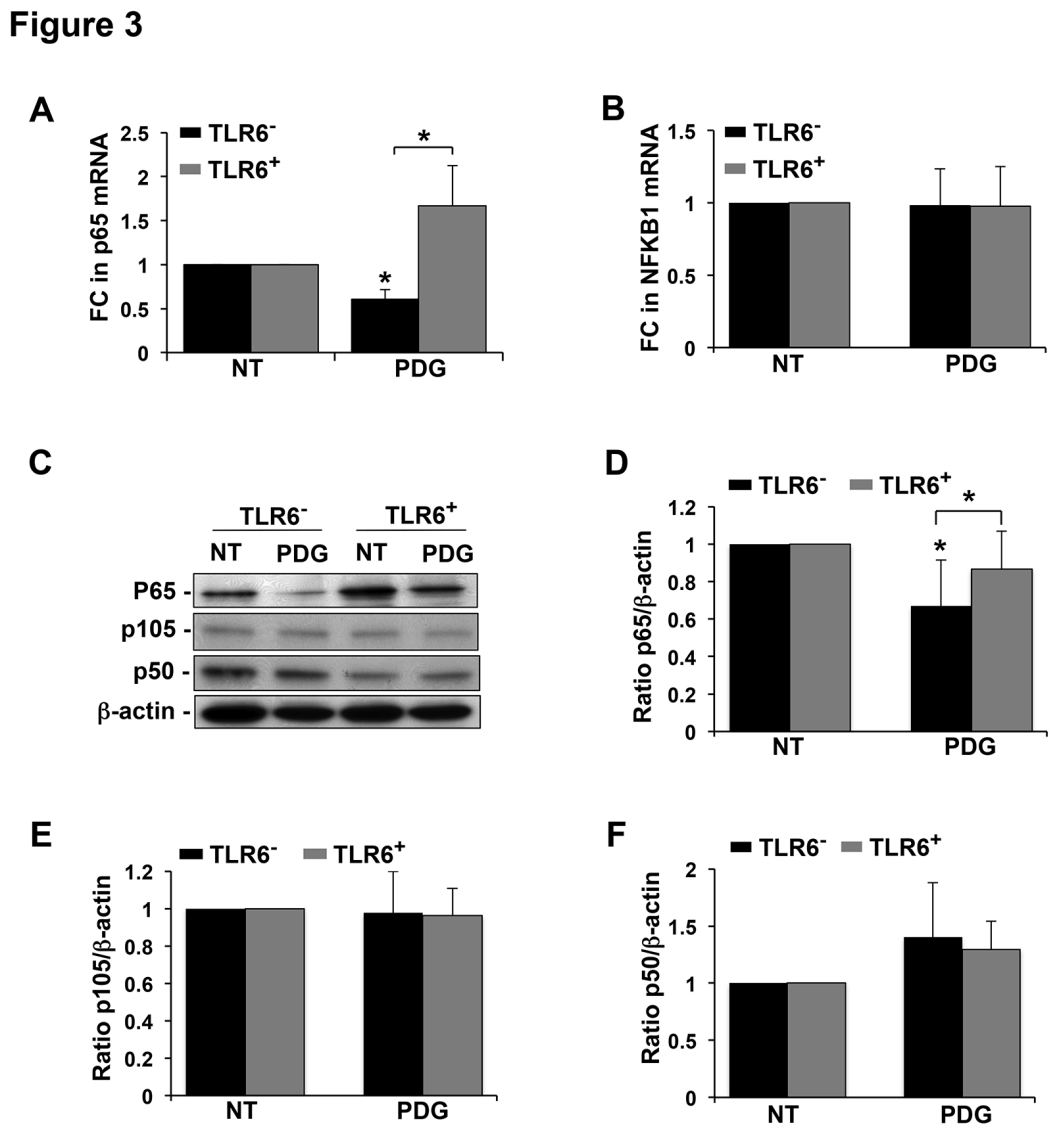
TLR6 restores PDG-mediated inhibition of trophoblast NF-κB p65 expression. TLR6^-^ or TLR6^+^ trophoblast cells were treated with no treatment (NT) or PDG (80μg/ml) for (A & B) 12h after which RNA was extracted, or (C - F) 48h, after which protein was collected. mRNA levels of (A) NF-κB p65 (n=4) and (B) NFKB1 (n=5) were measured by qRT-PCR. PDG treatment significantly reduced NF-κB p65, but not NFKB1, mRNA levels in the TLR6^-^ cells, and this was completely reversed by the presence of TLR6. (C) Protein levels of total NF-κB p65 and NFKB1 (p105 and p50) were analyzed by Western blot. A representative immunoblot is shown. Barcharts below show quantification of (D) p65, (E) p105, and (F) p50 protein expression as determined by densitometry with normalization to β-actin. PDG treatment significantly reduced NF-κB p65 (but not NFKB1 p105 or p50) protein levels in the TLR6^-^ cells, and this was reversed by the presence of TLR6 (n=3-5). *p<0.05 relative to the NT control unless otherwise indicated.

Classically, NF-κB represents the heterodimer of two subunits, p65 (RelA), and p50, which is derived from p105 (NFKB1) [35]. Upon activation, the NF-κB heterodimer translocates to the nucleus to regulate gene expression, and thus, major cellular functions [36]. Since we already reported that treatment of TLR6^-^ trophoblast cells with PDG reduced basal NF-κB activity levels and this was restored by the presence of TLR6, we next wanted to identify the specific NF-κB subunit involved in the regulation of PDG-mediated inhibition of IL-6 production and induction of apoptosis in the trophoblast [4]. We first assessed the expression of NF-κB p65 and NFKB1 mRNA. As shown in Figure 3A, treatment of TLR6^-^ trophoblast cells with PDG significantly reduced the expression of p65 mRNA by 44.4 ± 2.7 % compared to the NT control, and this was completely reversed by the presence of TLR6 (TLR6^+^). In contrast, PDG had no effect on the expression of NFKB1 mRNA in either the TLR6^-^ or TLR6^+^ trophoblast cells (Figure 3B). To further confirm the effect of PDG treatment on NF-κB p65 and NFKB1, western blot analysis was performed for total NF-κB p65 and NFKB1 p105 and p50 protein. Figure 3C, shows representative immunoblots for p65, p105, p50, and the loading control, β-actin. When densitometry was performed, it was found that PDG treatment of TLR6^-^ cells significantly reduced total p65 protein levels by 32.9 ± 24.4% when compared to the NT control, and this was significantly reversed by the presence of TLR6 (Figure 3D), similarly to our mRNA findings (Figure 3A). Also, in agreement with our gene expression data (Figure 3B), there were no significant changes in the protein levels of NFKB1 p105 (Figure 3E) or p50 (Figure 3F), in either the TLR6^-^ or the TLR6^+^ trophoblast cells after PDG treatment. 

### Identification of miR-329 as a potential regulator of trophoblast NF-κB p65 mRNA

**Figure 4:**
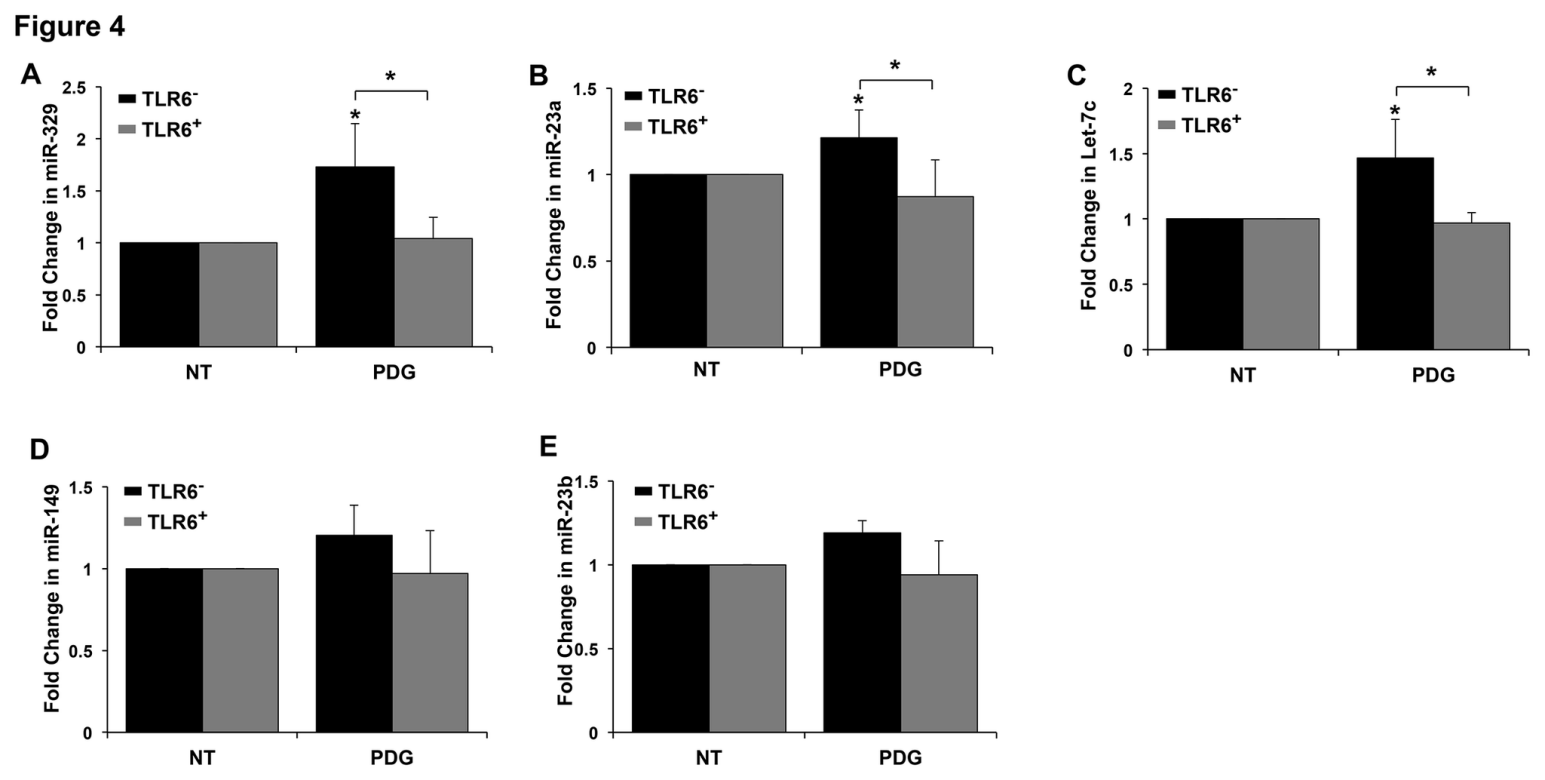
Regulation of miRs in PDG-treated trophoblast cells. TLR6^-^ or TLR6^+^ trophoblast cells were treated with no treatment (NT) or PDG (80μg/ml) for 12h, after which RNA was collected and the expression of: (A) miR-329; (B) miR-23a; (C) miR-let-7c; (D) miR-149; and (E) miR-23b was measured by qRT-PCR. Data are presented as fold change (FC) in miR expression after normalization to the endogenous control, miR-374. PDG treatment significantly upregulated trophoblast miR-329, miR-23a, miR-let-7c, and miR-23b expression in TLR6^-^, and this was significantly inhibited by the presence of TLR6 (TLR6^+^). *p<0.05 relative to the NT control unless otherwise indicated. Data are from 5-7 independent experiments.

Having established that PDG inhibited NF-κB p65 expression in TLR6^-^ but not TLR6^+^ cells, and that inhibition of NF-κB activity induced trophoblast apoptosis and inhibited IL-6 mRNA expression, similarly to the cell’s response to PDG, we next sought to determine the molecular mechanism involved. Since NF-κB p65 expression was inhibited at both the protein and mRNA level, we postulated that a miR which promotes mRNA instability might play a role in its regulation. In order to identify miRs that may regulate NF-κB p65 mRNA and are differentially expressed in PDG treated TLR6^-^ and TLR6^+^ cells, RNA from each group was subjected to a global miR microarray. Out of 1700 miRs that the cells were analyzed for, we identified 25 that are predicted to potentially target NF-κB p65 mRNA using the microrna.org algorithm. From these 25 miRs, trophoblast cells were found to express 8 at a detectable level: miR-7-5p; miR-186-5p; miR-155-5p; miR-22-3p; miR-185-5p; miR-138-5p; miR-329; and miR-362-3p (Table 1 and Table S1). Of these, only miR-155 and miR-329 were upregulated in PDG-treated TLR6^-^ cells and this response was reduced by the presence of TLR6 (Table 1). As shown in Table 1, PDG-treatment increased miR-329 expression by 1.85 fold in TLR6^-^ cells, while there was a 0.88 fold change in miR-329 expression in PDG-treated TLR6^+^ cells when compared to the NT controls. PDG-treatment increased miR-155 by 1.87 fold in TLR6^-^ cells, while PDG-treatment increased miR-155 by only 1.26 fold in TLR6^+^ cells. Having identified two miRs that were upregulated in PDG-treated TLR6^-^ cells and appeared differentially regulated in the TLR6^+^ cells, we next validated this by quantitative RT-PCR using specific primers. qRT-PCR revealed no significant difference in levels of miR-155 expression after treatment of TLR6^-^ cells with PDG when compared to the untreated control at any time point tested (Figure S1). However, as shown in Figure 4A, PDG treatment significantly increased the expression of miR-329 in TLR6^-^ cells by 1.73 ± 0.42 fold, while miR-329 expression was unchanged in PDG-treated TLR6^+^ cells. This finding validated the microarray data by showing that the presence of TLR6 significantly reversed PDG-induced miR-329 expression in the trophoblast (Table 1 & Figure 4A).

**Table 1:** Identification of miRs targeting NF-κB p65 mRNA through a global microRNA microarray.

		**TLR6^-^**			**TLR6^+^**		
**Predicted miRNA targeting NF-κB p65 mRNA**	**Expression level**	**NT**	**PDG**	**Fold Change**	**NT**	**PDG**	**Fold Change**
**Hsa-miR-155-5p**	High	554.8	1039.0	**1.873**	756.3	952.5	**1.259**
**Hsa-miR-329**	Low	191.1	352.7	**1.846**	390.2	342.5	**0.878**

TLR6^-^ and TLR6^+^ trophoblast cells were treated with either no treatment (NT) or PDG (80μg/ml) for 12h, after which total RNA was isolated. Table shows the miRs identified from the microarray analysis that are predicted to target NF-κB p65 mRNA, that were expressed at low-high levels, and that were upregulated in response to PDG in the TLR6^-^ cells but not or less in the TLR6^+^ cells. The data is represented as the signal intensity and fold change in miR expression between PDG-treated and NT cells.

### Identification of miR-23a and let-7c as a potential regulator of trophoblast IL-6 mRNA

After identifying miR-329 a potential regulator of NF-κB p65 mRNA expression, we next considered the possibility of the involvement of other miRs that can regulate IL-6 by directly targeting its mRNA. Again, using a global miR microarray, we identified 17 miRs that are putatively predicted to target IL-6 mRNA using the microrna.org algorithm and expressed by the trophoblast (Table 2 & Table S2). Of these 17 miRs, only 4 were expressed at low-high levels, were upregulated in PDG-treated TLR6^-^ cells, and differentially regulated in the PDG-treated TLR6^+^ cells: miR-23a; miR-23b; miR-149; and let-7c (Table 2). The expression of these 4 miRs were validated by quantitative RT-PCR using specific primers. PDG treatment of TLR6^-^ cells significantly increased the expression of miR-23a by 1.21 ± 0.16 fold and this was significantly reversed by the presence of TLR6 (Figure 4B). Similarly, PDG treatment of TLR6^-^ cells significantly increased the expression of let-7c by 1.46 ± 0.29 fold, while PDG had no effect on let-7c expression in PDG-treated TLR6^+^ cells (Figure 4C). However, in contrast to the microarray data, qRT-PCR showed no significant differences in miR-23b and miR-149 expression levels in PDG-treated TLR6^-^ and TLR6^+^ cells (Figure 4D & E). Thus, these findings only validated the microarray data showing that the presence of TLR6 significantly reversed PDG-induced miR-23a and let-7c expression in the trophoblast (Table 2 & Figure 4B & C).

**Table 2:** Identification of miRs targeting IL-6 mRNA through a global microRNA microarray.

		**TLR6^-^**			**TLR6^+^**		
**Predicted miRNA targeting IL-6 mRNA**	**Expression level**	**NT**	**PDG**	**Fold Change**	**NT**	**PDG**	**Fold Change**
**Hsa-miR-23a-3p**	High	10023.4	14600	**1.457**	8915.8	7762.7	**0.871**
**Hsa-miR-23b-3p**	High	8517.5	12156.7	**1.427**	7427.4	7176.3	**0.966**
**Hsa-let-7c**	Low	72.8	307.3	**4.224**	301.9	771.4	**2.555**
**Hsa-miR-149-3p**	Low	153	297	**1.936**	142	136	**0.961**

TLR6^-^ and TLR6^+^ trophoblast cells were treated with either no treatment (NT) or PDG (80μg/ml) for 12h, after which total RNA was isolated. Table shows the miRs identified from the microarray analysis that are predicted to target IL-6 mRNA, that were expressed at low-high levels, and that were upregulated in response to PDG in the TLR6^-^ cells but not or less in the TLR6^+^ cells. The data is represented as the signal intensity and fold change in miR expression between PDG-treated and NT cells.

### miR-329 regulates trophoblast apoptosis and NF-κB p65 and IL-6 expression

**Figure 5:**
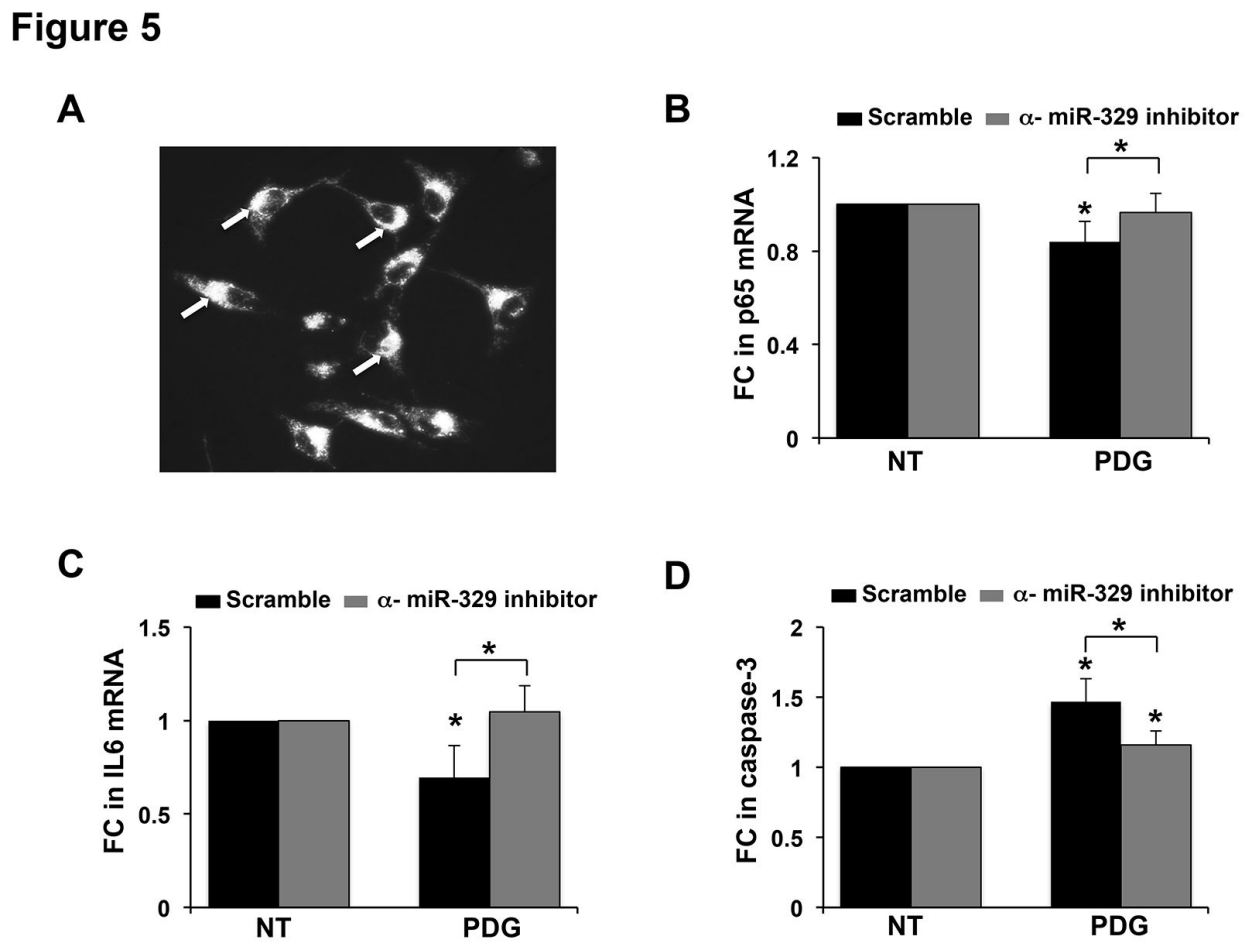
miR-329 regulates PDG-induced trophoblast apoptosis and inhibition of IL-6 expression by targeting NF-κB p65. (A) TLR6^-^ trophoblast cells were transfected with an anti-miR-inhibitor scramble sequence labeled with Cy3. After 24h, uptake efficiency was evaluated by fluorescent microscopy and a 99% transfection efficiency was observed. (B - D) TLR6^-^ trophoblast cells were transfected with either an anti-miR scramble sequence or a specific anti-miR-329 inhibitor. Post-transfection, cells were treated with either NT or PDG (80μg/ml) for 12h, after which (B) NF-κB p65 mRNA and (C) IL-6 mRNA levels were measured by qRT-PCR. (D) After 48h caspase-3 activity was measured. Treatment of scramble control cells with PDG significantly reduced NF-κB p65 and IL-6 mRNA expression levels and upregulated caspase-3 activity, and these responses were significantly reversed by the presence of the anti-miR-329 inhibitor. *p<0.05 relative to the NT control unless otherwise indicated (n=3-5).

Since PDG upregulated trophoblast miR-329, which is predicted to target NF-κB p65, we hypothesized that miR-329 through this mechanism, might be regulating TLR2-mediated apoptosis and IL-6 mRNA expression. To test this, TLR6^-^ cells were transfected with a specific anti-miR-329 inhibitor or a scramble control sequence, followed by treatment with or without PDG. The anti-miR-329 inhibitor significantly reduced miR-329 expression when compared to the scramble control sequence (data not shown). As shown in Figure 5A, a 99% transfection efficiency was achieved. Treatment of scramble control cells with PDG significantly decreased NF-κB p65 mRNA levels. In contrast, PDG treatment of the cells transfected with the anti-miR-329 inhibitor had no effect on NF-κB p65 expression (Figure 5B). Similarly, PDG treatment significantly decreased IL-6 mRNA levels in the scramble control cells, while in the anti-miR-329 inhibitor cells, there was no effect on IL-6 mRNA (Figure 5C). Lastly, while exposure to PDG significantly elevated caspase-3 activity in the scramble control cells, the presence of the anti-miR-329 inhibitor significantly prevented this response (Figure 5D).

### Combined inhibition of miR-23a and let-7c regulates trophoblast IL-6 expression

**Figure 6:**
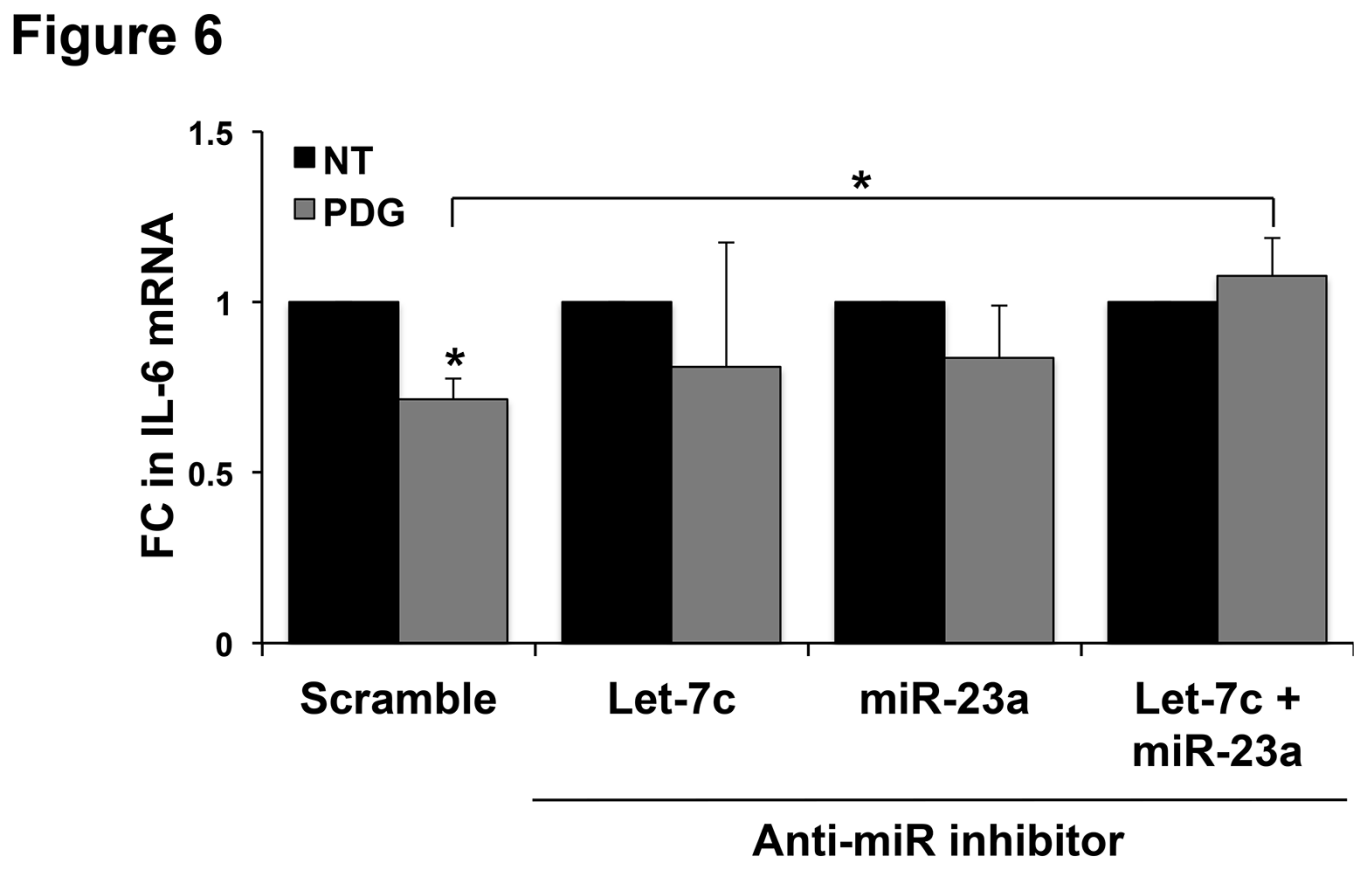
miR-23a and miR-let-7c regulate PDG-mediated inhibition of IL-6 expression. TLR6^-^ trophoblast cells were transfected with an either: an anti-miR scramble sequence; a specific anti-miR-23a inhibitor; a specific anti-let-7c inhibitor; or both the inhibitors of miR-23a and let-7c. Post-transfection, cells were treated with either no treatment (NT) or PDG (80μg/ml) for 12h, after which IL-6 mRNA levels were measured by qRT-PCR. Treatment of scramble control cells with PDG significantly reduced IL-6 mRNA levels, and these responses were significantly reversed by the presence of both the anti-miR-23a and anti-let-7c inhibitor. *p<0.05 relative to the NT control unless otherwise indicated (n=5).

Since PDG-treatment upregulated trophoblast expression of miR-23a and let-7c, miRs that putatively target IL-6 mRNA, we hypothesized that miR-let-7c or miR23a through this mechanism, might also be regulating PDG-mediated inhibition of IL-6 expression. To test this, TLR6^-^ cells were transfected with a specific anti-miR-23a inhibitor, an anti-let-7c inhibitor, or a combination of both, followed by treatment with or without PDG. The anti-Let-7c and anti-miR-23a inhibitors reduced Let-7c and miR-23a expression, respectively, when compared to the scramble control sequence (data not shown). As shown in Figure 6, treatment of scramble control cells with PDG significantly decreased IL-6 mRNA levels. In contrast, PDG treatment of the cells transfected with either the anti-Let-7c inhibitor alone, the anti-miR-23a inhibitor alone, or a combination of both the anti-let-7c and anti-miR-23a inhibitors, had no significant effect on IL-6 mRNA levels. Moreover, the combination of the miR-23a and let-7c inhibitors significantly reversed the PDG-inhibition of IL-6 mRNA when compared to PDG-treated scramble control cells (Figure 6). This data suggests that the combined inhibition of miR-23a and let-7c is necessary for restoring PDG-mediated inhibition of IL-6 expression in trophoblast. 

## Discussion

Innate immune sensors, such as Toll-like receptors (TLRs), have been shown to play a role in the pathogenesis of infection-associated pregnancy complications by mediating immune responses at the maternal-fetal interface [1,4]. Classically, TLR activation generates an inflammatory response, however, TLR2, TLR3, and TLR4 can also induce apoptosis in various cell types [4,10,37-41]. Therefore, both TLR-mediated inflammation and apoptosis at the maternal-fetal interface may be key factors in triggering placental injury and adverse pregnancy outcome [4]. Indeed, studies from our laboratory have previously demonstrated that administration of gram-positive bacterial PDG to pregnant mice induces placental apoptosis [4]. We have also demonstrated that PDG induces apoptosis in human first trimester trophoblast cells that constitutively express TLR2, and its co-receptors, TLR1 and TLR10, but that lack the co-receptor TLR6 [4,11,15]. This apoptotic response was previously found to be associated with reduced constitutive IL-6 protein secretion and reduced NF-κB activity [4,15]. Furthermore, the ectopic expression of TLR6 in first trimester trophoblast cells prevents PDG-induced apoptosis and restores the cell’s IL-6 production [4]. This study suggested that activation of TLR2 by bacterial PDG induces a differential response to be generated by the trophoblast in the presence of different co-receptors. Indeed, the induction of apoptosis by PDG is dependent upon TLR1, TLR2, and TLR10 [4,11,15]. Moreover, in third trimester trophoblast cells, which constitutively express TLR6, no apoptosis in response to PDG is observed, and instead there is cytokine production [42]. Thus, TLR6 appears to provide a cellular switch that regulates cell survival and chemokine production in the trophoblast. In this current study we have investigated the molecular mechanism by which TLR2 and TLR6 regulate trophoblast apoptosis and IL-6 production. Herein we report for the first time that TLR2 activation by PDG induces miR-329 expression, which plays a pivotal role in regulating PDG-induced trophoblast apoptosis and inhibition of IL-6 expression by targeting the NF-κB subunit, p65. In addition to miR-329, we have identified 2 miRs, miR-23a and let-7c, that appear to regulate trophoblast IL-6 expression by directly targeting its mRNA.

Using our model of TLR6 expressing (TLR6^+^) and deficient (TLR6^-^) human first trimester trophoblast cells, in this current study we first questioned whether PDG-induced suppression of IL-6 protein secretion [4] was regulated at the transcriptional level, and whether this and the associated induction of apoptosis was under the regulation of NF-κB. We found that PDG-treatment of TLR6^-^ cells inhibited IL-6 mRNA expression and this could be prevented by the presence of TLR6. Furthermore, inhibition of NF-κB activity in the TLR6^-^ cells recapitulated the response triggered by PDG; reduced IL-6 mRNA expression and elevated caspase-3 activity [4,11,15]. Thus, it appears that the inhibition of NF-κB activity following TLR2 activation by PDG is what is responsible for the induction of trophoblast apoptosis and inhibition of IL-6 production. These results are consistent with previous studies in various model systems showing that inhibition of NF-κB activity induces apoptosis and inhibits IL-6 production in other cell types [43-45]. Moreover, using epithelial cells and monocytes Aliprantis et al., demonstrated that TLR2 mediates apoptosis via Fas-associated death domain (FADD) binding to MyD88, and subsequent activation of the caspase pathway [37,46]; much like we see in the trophoblast [11]. Furthermore, inhibition of NF-κB activation downstream of MyD88 facilitates TLR2-mediated apoptosis [46]. 

Next we questioned precisely how TLR2 activation by PDG was inhibiting basal NF-κB activity in the trophoblast, and the underlying molecular mechanism involved. We first established that PDG treatment of TLR6^-^ cells was inhibiting NF-κB activity by decreasing the expression of the NF-κB p65 submit both at the mRNA and protein level, while the expression of NFKB1 mRNA and protein (p105 and p50) was unaffected. In contrast, NF-κB p65 expression levels were unaffected in PDG-treated TLR6^+^ cells. These data suggest that following TLR2 activation in the absence of TLR6, a factor was either preventing NF-κB p65 transcription, or promoting its degradation. We postulated that the latter might be occurring through a microRNA, since miRs can regulate gene expression through destabilization [16,17], and a number of miRs are known to be induced following TLR activation [17]. Through a global microRNA microarray, bioinformatics databases, and quantitative RT-PCR, we identified miR-329 as a novel regulator of NF-κB p65 mRNA that was differentially expressed in PDG-treated TLR6^-^ and TLR6^+^ trophoblast cells. Following treatment with PDG, miR-329 expression was upregulated in TLR6^-^ cells and this was completely prevented in the presence of TLR6. Furthermore, a specific miR-329 inhibitor prevented PDG-mediated inhibition of NF-κB p65 and IL-6 mRNA expression, and inhibited PDG-induced apoptosis in the TLR6^-^ trophoblast cells. To date, little is known about the function of miR-329, or its targets. However, low expression levels of miR-329 correlate with survival in glioblastoma multiforme patients [47], studies of hippocampal neurons suggest it to be involved in dendritic outgrowth [48], and miR-329 expression is decreased in airway smooth muscle treated with IL-1β, TNFα, and IFNγ [49]. We believe this study to be the first report that TLR2 activation by PDG induces miR-329 expression, and that miR-329 appears to target NF-κB p65 mRNA.

Having identified miR-329 as a regulator of trophoblast IL-6 expression through the NF-κB p65 pathway, we also considered the involvement of additional miRs that might regulate the PDG-mediated IL-6 response by directly targeting IL-6 mRNA. Again, using a global microRNA microarray, bioinformatics databases, and quantitative RT-PCR, we identified miR-23a and let-7c as potential regulators of IL-6 that were differentially expressed in PDG-treated TLR6^-^ and TLR6^+^ trophoblast cells. Following treatment with PDG, miR-23a and miR-let-7c expression were both upregulated in TLR6^-^ cells and this was completely prevented by the presence of TLR6. Furthermore, by inhibiting both miR-23a and let-7c simultaneously, PDG-mediated inhibition of IL-6 expression could be prevented. This finding highlights the functional redundancy that occurs between miRs [50]. While IL-6 can induce miR-23a in hepatocytes [51], and miR-23a targets IL-6R in gastric adenocarcinoma cells [52], to our knowledge, there is no information regarding the regulation of IL-6 by miR-23a. Similarly, while let-7 family members regulate oncogene, cell cycle, proliferation, apoptosis and immune responses to pathogens [53-55], and let-7a has been reported to directly target IL-6 mRNA by binding its 3’UTR [56], nothing has been reported for let-7c in terms of regulating IL-6 expression, although it has been shown to target IL-10 in PBMCs [57]. These studies suggest the important role of miR-23a and miR-let-7c in the regulation of various cellular functions; however, we believe that our studies are first to indicate that TLR2 activation by PDG induces miR-23a and miR-let-7c expression which in turn regulates IL-6 mRNA expression.

In summary, we have identified miRs that regulate TLR2 mediated responses in human trophoblast. Our findings suggest that miR-329 plays a pivotal role in regulating human first trimester trophoblast cell responses to PDG by targeting the NF-κB p65 subunit, leading to apoptosis and inhibition of IL-6 expression, and TLR6 reverses this cellular response by preventing the TLR2-mediated induction of miR-329. In addition, miR-23a and let-7c regulate TLR2-mediated inhibition of IL-6 expression by directly targeting IL-6 mRNA. Thus, miR-329, miR-23a and let-7c provide a novel molecular mechanism that regulates trophoblast TLR2/TLR6 function in response to gram-positive bacterial components.

## Supporting Information

Figure S1 **Expression of miR-155 in PDG-treated trophoblast cells.** TLR6^-^ trophoblast cells were treated with no treatment (NT) or PDG (80μg/ml) for 3h, 6h, 12h, or 24hr after which RNA was collected and the expression of miR-155 was measured by qRT-PCR. Data are presented as fold change (FC) in miR expression after normalization to the endogenous control, miR-374. Treatment with PDG had no effect on the expression levels of miR-155.
(TIF)

Table S1 **Identification of additional miRs targeting NF-κB p65 mRNA through a global microRNA microarray.** TLR6^-^ and TLR6^+^ trophoblast cells were treated with either no treatment (NT) or PDG (80μg/ml) for 12h, after which total RNA was isolated. Table shows the additional miRs identified from the microarray analysis that are predicted to target NF-κB p65 mRNA. The data is represented as the signal intensity and fold change in miR expression between PDG-treated and NT cells.
(PDF)

Table S2 **Identification of additional miRs targeting IL-6 mRNA through a global microRNA microarray.** TLR6^-^ and TLR6^+^ trophoblast cells were treated with either no treatment (NT) or PDG (80μg/ml) for 12h, after which total RNA was isolated. Table shows the the additional miRs identified from the microarray analysis that are predicted to target IL-6 mRNAs. The data is represented as the signal intensity and fold change in miR expression between PDG-treated and NT cells.
(PPTX)
