# Supplementary figures and images for: Identification of microRNAs That Regulate TLR2-Mediated Trophoblast Apoptosis and Inhibition of IL-6 mRNA
Source: PLoS One. 2013 Oct 15;8(10):e77249. doi: 10.1371/journal.pone.0077249 (PMC3797072; doi:10.1371/journal.pone.0077249)

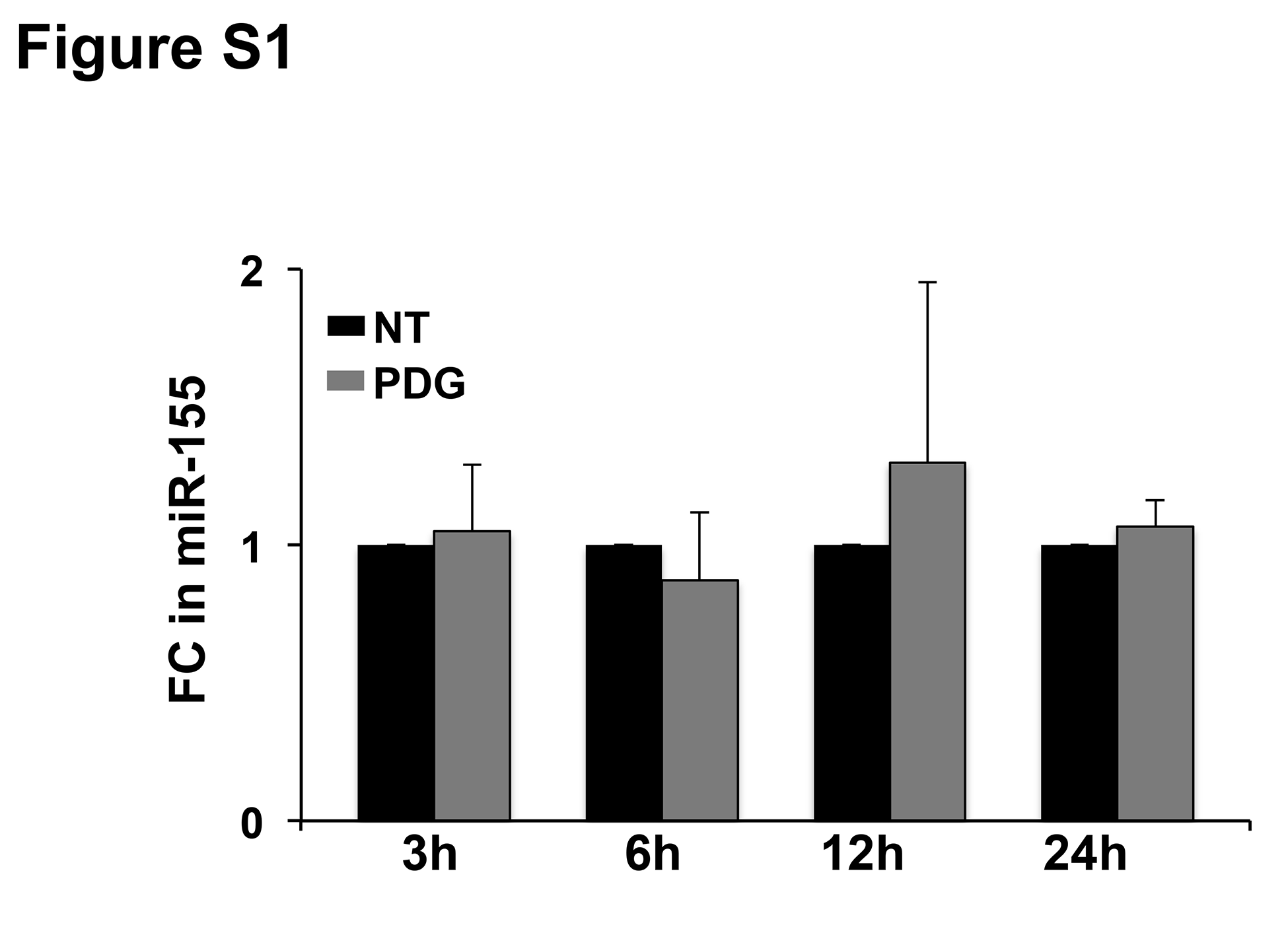

Supplement: Figure S1 — Expression of miR-155 in PDG-treated trophoblast cells. TLR6- trophoblast cells were treated with no treatment (NT) or PDG (80μg/ml) for 3h, 6h, 12h, or 24hr after which RNA was collected and the expression of miR-155 was measured by qRT-PCR. Data are presented as fold change (FC) in miR expression after normalization to the endogenous control, miR-374. Treatment with PDG had no effect on the expression levels of miR-155. (TIF) [file pone.0077249.s001.tif]
