# Supplementary material for: Identification of microRNAs That Regulate TLR2-Mediated Trophoblast Apoptosis and Inhibition of IL-6 mRNA
Source: PLoS One. 2013 Oct 15;8(10):e77249. doi: 10.1371/journal.pone.0077249 (PMC3797072; doi:10.1371/journal.pone.0077249)
Supplement: Table S1 — Identification of additional miRs targeting NF-κB p65 mRNA through a global microRNA microarray. TLR6- and TLR6+ trophoblast cells were treated with either no treatment (NT) or PDG (80μg/ml) for 12h, after which total RNA was isolated. Table shows the additional miRs identified from the microarray analysis that are predicted to target NF-κB p65 mRNA. The data is represented as the signal intensity and fold change in miR expression between PDG-treated and NT cells. (PDF) [file pone.0077249.s002.pdf]

Table S1

Predicted miRNA targeting NF-κB p65 mRNA	Expression level	TLR6⁻ NT	TLR6⁻ PDG	TLR6⁻ Fold change	TLR6⁺ NT	TLR6⁺ PDG	TLR6⁺ Fold change
**Hsa-miR-7-5p**	High	1,759.5	1,525.3	**0.867**	1,666.6	1,572.4	**0.943**
**Hsa-miR-186-5p**	High	1,490.9	1,034.8	**0.694**	1,822.1	1,475.8	**0.810**
**Hsa-miR-22-3p**	High	7,102.8	5,079.6	**0.715**	5,847.1	5,583.8	**0.955**
**Hsa-miR-185-5p**	Low	532.7	440.3	**0.826**	467.1	491.6	**1.052**
**Hsa-miR-362-3p**	Low	173.4	109.9	**0.634**	156.5	171.9	**1.099**
**Hsa-miR-138-5p**	Very Low	117.0	84.0	**0.718**	99.7	122.2	**1.225**
