# Supplementary material for: Identification of microRNAs That Regulate TLR2-Mediated Trophoblast Apoptosis and Inhibition of IL-6 mRNA
Source: PLoS One. 2013 Oct 15;8(10):e77249. doi: 10.1371/journal.pone.0077249 (PMC3797072; doi:10.1371/journal.pone.0077249)
Supplement: Table S2 — Identification of additional miRs targeting IL-6 mRNA through a global microRNA microarray. TLR6- and TLR6+ trophoblast cells were treated with either no treatment (NT) or PDG (80μg/ml) for 12h, after which total RNA was isolated. Table shows the the additional miRs identified from the microarray analysis that are predicted to target IL-6 mRNAs. The data is represented as the signal intensity and fold change in miR expression between PDG-treated and NT cells. (PPTX) [file pone.0077249.s003.pptx]

## Slide 1
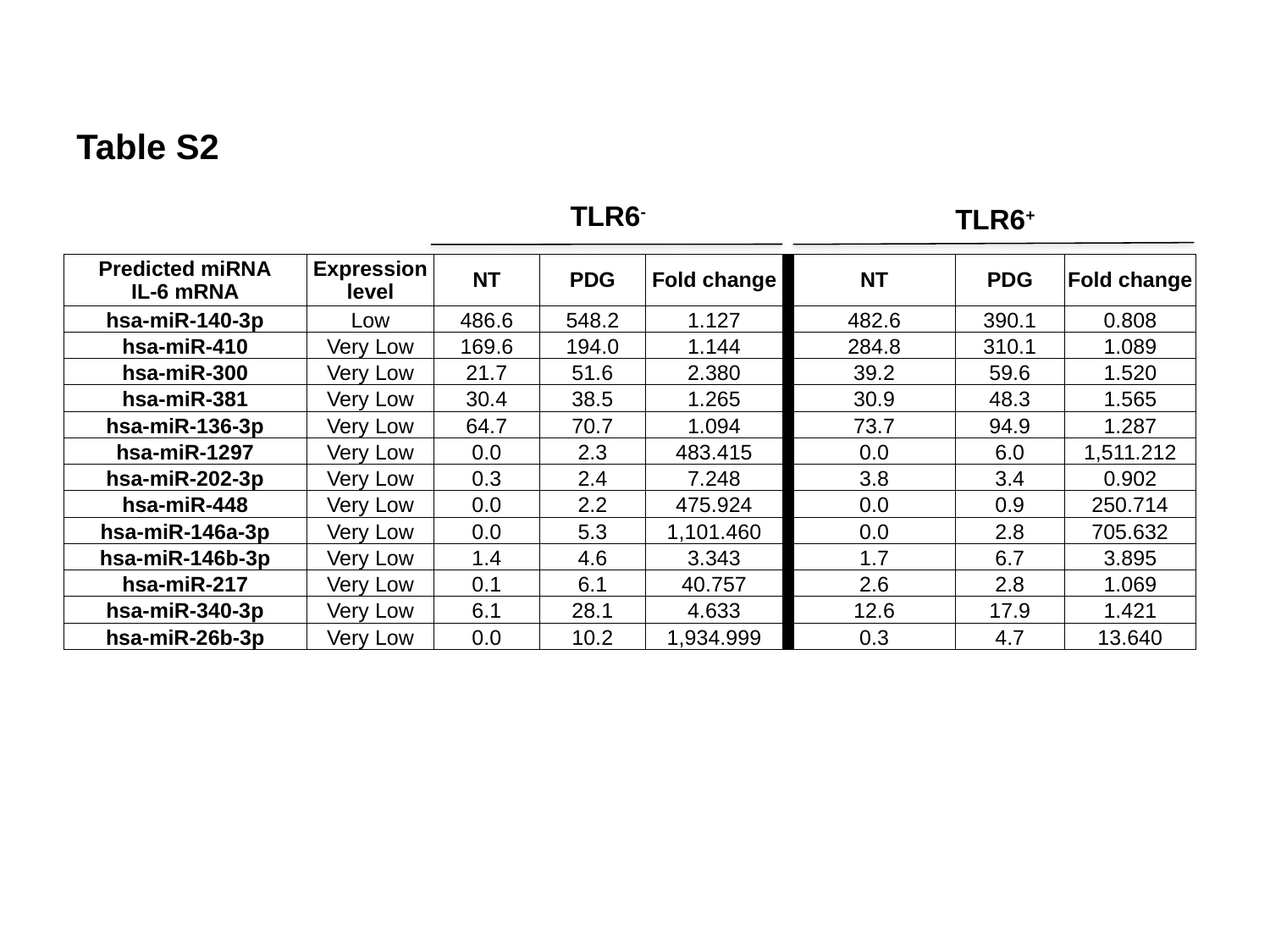

Table S2
TLR6-
TLR6+
| Predicted miRNA IL-6 mRNA | Expression level | NT | PDG | Fold change | | NT | PDG | Fold change |
| --- | --- | --- | --- | --- | --- | --- | --- | --- |
| hsa-miR-140-3p | Low | 486.6 | 548.2 | 1.127 | | 482.6 | 390.1 | 0.808 |
| hsa-miR-410 | Very Low | 169.6 | 194.0 | 1.144 | | 284.8 | 310.1 | 1.089 |
| hsa-miR-300 | Very Low | 21.7 | 51.6 | 2.380 | | 39.2 | 59.6 | 1.520 |
| hsa-miR-381 | Very Low | 30.4 | 38.5 | 1.265 | | 30.9 | 48.3 | 1.565 |
| hsa-miR-136-3p | Very Low | 64.7 | 70.7 | 1.094 | | 73.7 | 94.9 | 1.287 |
| hsa-miR-1297 | Very Low | 0.0 | 2.3 | 483.415 | | 0.0 | 6.0 | 1,511.212 |
| hsa-miR-202-3p | Very Low | 0.3 | 2.4 | 7.248 | | 3.8 | 3.4 | 0.902 |
| hsa-miR-448 | Very Low | 0.0 | 2.2 | 475.924 | | 0.0 | 0.9 | 250.714 |
| hsa-miR-146a-3p | Very Low | 0.0 | 5.3 | 1,101.460 | | 0.0 | 2.8 | 705.632 |
| hsa-miR-146b-3p | Very Low | 1.4 | 4.6 | 3.343 | | 1.7 | 6.7 | 3.895 |
| hsa-miR-217 | Very Low | 0.1 | 6.1 | 40.757 | | 2.6 | 2.8 | 1.069 |
| hsa-miR-340-3p | Very Low | 6.1 | 28.1 | 4.633 | | 12.6 | 17.9 | 1.421 |
| hsa-miR-26b-3p | Very Low | 0.0 | 10.2 | 1,934.999 | | 0.3 | 4.7 | 13.640 |
